# Lactylation and tumor immune regulation: insights from recent studies

**DOI:** 10.3389/fimmu.2025.1699314

**Published:** 2026-01-13

**Authors:** Chunhong Li, Xiulin Jiang, Yixiao Yuan, Qiang Wang

**Affiliations:** 1 Department of Oncology, Suining Central Hospital, Suining, Sichuan, China; 2 Department of Systems Biology, City of Hope Comprehensive Cancer Center Biomedical Research Center, Monrovia, CA, United States; 3 Department of Gastrointestinal Surgical Unit, Suining Central Hospital, Suining, Sichuan, China

**Keywords:** immune evasion, immunotherapy, lactylation, tumor immunity, tumor microenvironment

## Abstract

Lactate, a major product of glycolysis, accumulates abundantly in the tumor microenvironment (TME), serving not only as a hallmark of metabolic dysregulation but also as a key driver of immunosuppression. In recent years, lysine lactylation (Kla), a novel post-translational modification (PTM), has been identified, linking lactate metabolism closely with epigenetic regulation. Current studies indicate that lactylation modulates gene transcription and metabolic pathways in tumor cells while broadly influencing immune cell functions. For example, histone lactylation in macrophages promotes M2 polarization, enhancing immunosuppressive phenotypes; T cells, natural killer (NK) cells, dendritic cells (DCs), and myeloid-derived suppressor cells (MDSCs) may also be regulated by lactylation, thereby affecting anti-tumor immune responses and the efficacy of immune checkpoint inhibitors. As the mechanistic understanding of lactylation deepens, its roles in tumor immune evasion and therapy resistance are becoming increasingly evident. Targeting lactate metabolism and lactylation-related enzymatic processes, potentially in combination with immunotherapy, may represent a promising therapeutic strategy. This mini-review summarizes recent advances in lactylation research in tumor immunity and discusses its potential clinical implications and future directions.

## 1 Introduction

Post-translational modifications (PTMs) are central mechanisms for regulating protein function and play critical roles in cellular signal transduction, metabolic homeostasis, gene transcription, and immune responses (1). Accumulating evidence has shown that aberrant PTMs are not only closely associated with tumorigenesis but also directly modulate the tumor immune microenvironment, thereby influencing anti-tumor immunity and therapeutic responsiveness (2). Classical PTMs, such as acetylation, methylation, and phosphorylation, have been extensively studied, while the discovery of novel modifications continues to expand our understanding of epigenetic regulation (3, 4).

In 2019, lysine lactylation (Kla) was first identified, providing new evidence for the crosstalk between metabolism and epigenetics (5). Lactylation is a lysine side-chain modification using lactate as a donor and can directly participate in chromatin remodeling and gene transcriptional regulation (6). This finding challenges the traditional view of lactate as merely a glycolytic byproduct and reveals its novel role in cell fate determination and immune regulation. Metabolic reprogramming, particularly enhanced glycolysis known as the Warburg effect, is a hallmark of tumors, leading to excessive lactate accumulation in the TME (7, 8). Elevated lactate not only alters the pH and redox state of the TME but also influences epigenetic regulation in diverse immune cells through lactylation (9). For instance, lactylation promotes tumor-associated macrophages (TAMs) toward an immunosuppressive M2 phenotype and may impair T cell effector functions, facilitating tumor immune evasion (10).

Increasing evidence suggests that lactylation serves as a critical nexus linking metabolic homeostasis, epigenetic regulation (11–13), and tumor immune responses (14). Understanding lactylation not only broadens the scope of tumor immunology research but also provides potential targets for novel therapeutic strategies. This review summarizes current advances in lactylation research in tumor immunity, with a focus on its roles in immune cell regulation, immune evasion mechanisms, and immunotherapy responses, and outlines future research directions and translational potential.

Unlike previous reviews that primarily focus on the role of protein lactylation in tumor initiation and progression, our mini-review, “Lactylation and Tumor Immune Regulation: Insights from Recent Studies,” specifically emphasizes the impact of lactylation on tumor immune regulation. We highlight the latest research advances elucidating how lactylation modulates immune cell function, the tumor microenvironment, and immune evasion. Moreover, we discuss the therapeutic potential of targeting lactylation to overcome immunosuppression and enhance the efficacy of cancer immunotherapy, offering a perspective that extends beyond traditional tumor biology to translational applications in tumor immunotherapy.

## 2 Regulatory mechanisms of lactylation

The concept of lactylation emerged from investigations into the relationship between cellular metabolism and histone modifications (15). Previous studies have shown that various metabolic intermediates, such as acetyl-CoA and succinyl-CoA, can serve as donors for histone acylation, thereby regulating gene expression. Within this context, Zhang et al. (5) first predicted and experimentally validated lysine lactylation (Kla) as a novel histone PTM (16, 17). Using high-resolution mass spectrometry, lactyl groups were detected on core histone lysine residues, confirming the presence of lactylation (18, 19). Further, through the development of pan-anti-lactyllysine antibodies, multiple histone lactylation sites were identified in human MCF-7 breast cancer cells and mouse bone marrow-derived macrophages (20). Crucially, isotope tracing experiments demonstrated that intracellular lactate could directly contribute to lysine lactylation, establishing a direct link between lactate metabolism and epigenetic modification (10). This discovery not only revealed a new function for lactate in gene transcription but also suggested its potential roles in physiological homeostasis and pathological conditions, particularly tumor progression and immune responses.

It is noteworthy that the enzymatic mechanisms of lactylation remain incompletely understood. Studies suggest that acetyltransferases such as p300/CBP may possess catalytic potential, whereas the existence and functions of lactylation “erasers” are still under investigation (21, 22). Thus, lactylation represents a key new node in metabolic-epigenetic crosstalk, and mechanistic studies are expected to provide crucial insights into tumor development and immune regulation. PTMs, biochemical modifications occurring post-protein synthesis, dynamically modulate protein activity, localization, and molecular interactions through the addition of functional groups such as phosphate, methyl, and acetyl groups, thereby expanding protein functional diversity. Lysine acetylation (Kac), a widely studied PTM, is reversible, evolutionarily conserved, and tightly regulated (23). Kac regulation involves “writers,” “readers,” and “erasers,” a paradigm now extended to other lysine acylations, including succinylation, butyrylation, propionylation, malonylation, glutarylation, 2-hydroxyisobutyrylation, and β-hydroxybutyrylation. As a novel lysine acylation, lactylation similarly involves specific “writers” and “erasers,” though its “readers” remain unidentified. Study identifies HBO1 as a lysine lactyltransferase that directly mediates histone lactylation and regulates transcription. HBO1 catalyzes Kla both *in vitro* and in cells, with E508 as the critical active site, and predominantly modifies histones such as H3K9la, enhanced by cofactors like JADE1 and BRPF2 (24). Mechanistically, HBO1-dependent H3K9la is enriched at transcription start sites (TSSs), driving gene expression that supports key signaling pathways and tumorigenesis (24).

### 2.1 Histone lactylation

The molecular mechanisms of protein lactylation involve two fundamentally distinct but complementary modes-an enzyme-mediated “precision writing” process and a metabolism-driven “global splashing” effect-that together shape the complex lactylation landscape. On the one hand, the enzymatic pathway functions like a finely tuned writer (25). Histone acetyltransferase p300 acts as the primary “writer,” using lactyl-CoA rather than lactate itself as the direct donor to covalently attach lactyl groups to specific lysine residues (such as H3K18la) (26). Lactyl-CoA can be generated through ATP-dependent synthesis by AARS1 or potentially via mitochondrial intermediates that remain under investigation[ (27){Zong, 2024 #6464]. Histone acetyltransferases (HATs) exhibit broad acyltransferase activity and are classified into p300/CBP, GNAT, and MYST families based on sequence and structural homology, with p300 being the most versatile (28, 29). Zhao et al. demonstrated that p300 can function as a histone lactylation “writer,” catalyzing the transfer of lactyl groups from L-lactyl-CoA to histones (30). Yang et al. further confirmed the role of p300 in histone lactylation during cholangiocarcinoma pathogenesis. HBO1, a MYST family member, also regulates histone lactylation: overexpression of HBO1 in HeLa and HEK-293T cells increases histone lactylation levels, while knockout reduces it; *in vitro* assays confirm HBO1’s direct catalytic activity (31). Aminoacyl-tRNA synthetases AARS1/2 act as lactate sensors in mammalian cells and mediate lactylation of non-histone substrates such as cGAS, suppressing innate immune responses and affecting viral infection and immune evasion (32). Recent studies have shown that nuclear GTPSCS can function as a lactoyl-CoA synthetase, with GTPSCS/p300 co-regulating histone H3K18la and GDF15 expression, thereby promoting glioma proliferation and radioresistance (33).

Similar to histone acetyltransferases, histone deacetylases (HDACs) can remove non-acetyl acyl groups. *In vitro*, class I HDACs (HDAC1-3) and class III sirtuins (SIRT1-3) significantly reduce histone L-lactylation, with HDAC1–3 being the most effective lysine delactylases (23, 29, 34). HDAC1–3 can also remove D-lactylation from non-histone proteins, showing higher efficiency for D-lactylation than L-lactylation. In cells, HDAC1 and HDAC3 are the major “erasers” of histone lactylation (23, 29, 34). Collectively, p300 and HBO1 mediate lactylation “writing,” whereas SIRT1–3 and HDAC1–3 mediate its removal.

### 2.2 Non-histone lactylation

Non-histone lactylation is similarly regulated by p300, SIRT3, and HDAC3 (35). Gao et al. identified 9,275 lactylation sites in hepatocellular carcinoma (HCC), of which 9,256 were non-histone (36). Correlation analysis between lactylation levels and expression of p300 and HDAC1–11 indicated a positive association with HDAC1-3. Functional experiments showed that p300 inhibition reduces lactylation, while HDAC3 knockdown increases it, confirming their roles as “writer” and “eraser” in non-histone substrates. Jin et al. reported that SIRT3 acts on non-histone proteins, including cyclin E2, reducing their lactylation levels, and crystallography confirmed SIRT3’s delactylase activity. Lysine acetyltransferase 8 (KAT8) also functions as a pan-lactylation “writer,” catalyzing lactylation of multiple proteins involved in diverse biological processes (37). Interestingly, some non-enzymatic lactylation occurs independently of enzymatic catalysis, primarily targeting glycolytic enzymes (38, 39). Lactylation inhibits their activity, thereby downregulating glycolysis. This process uses methylglyoxal, a glycolytic byproduct, as a precursor, which is converted to S-D-lactylglutathione by glyoxalase 1. S-D-lactylglutathione directly donates lactyl groups to lysine residues, completing lactylation without enzymatic involvement (40).

Lactylation is a lysine-specific post-translational modification that occurs on both histone and non-histone proteins, serving as a key metabolic–epigenetic regulatory mechanism (41, 42). Histone lactylation, such as at H3K18 and H3K23, modulates chromatin accessibility and transcription, thereby influencing immune cell functions including T cell exhaustion, regulatory T cell activation, and tumor-associated macrophage polarization (43, 44). Non-histone lactylation targets critical regulatory proteins, altering their interactions and activity; notable examples include MOESIN K72 in Tregs, NBS1 K388 in the MRN complex for homologous recombination, and XLF K288 within its Ku-binding motif for non-homologous end joining (45). Other non-histone lactylation targets, such as ROCK1, PKM2, and HMGB1, contribute to NK cell cytotoxicity, macrophage polarization, and inflammatory responses (29, 46). Collectively, lactylation at histone and non-histone lysine residues links glycolysis-derived lactate to immune regulation, DNA repair, and therapy resistance, highlighting its central role in tumor biology and potential as a therapeutic target.

## 3 Lactylation in the tumor microenvironment

Metabolic reprogramming is a hallmark of cancer, with one of the most prominent features being sustained glycolytic activity (47). Even under aerobic conditions, tumor cells preferentially generate energy via glycolysis, a phenomenon known as the Warburg effect (48). This metabolic phenotype leads to substantial accumulation of lactate in the TME, creating a milieu characterized by high lactate, hypoxia, and acidity. Historically regarded as a mere byproduct of glycolysis, lactate is now recognized as a signaling molecule and epigenetic regulator following the discovery of lactylation.

Lactylation establishes a novel bridge between metabolism and epigenetics (41). Elevated intracellular lactate in tumor cells drives histone lactylation, thereby regulating gene expression to promote adaptation to hypoxia and nutrient deprivation, enhancing proliferation and metastatic potential (14). Beyond its effects on tumor cells, high lactate levels in the TME also modulate immune cell function via lactylation (7). For instance, histone lactylation in macrophages skews polarization toward the immunosuppressive M2 phenotype, while T cell effector function may be impaired due to lactate-induced transcriptional remodeling, resulting in diminished cytotoxicity and increased exhaustion (49). Therefore, lactylation in the TME exerts a dual effect: it promotes malignant progression through tumor-intrinsic gene regulation and suppresses anti-tumor immune responses by reshaping the immune microenvironment. This metabolic-epigenetic interplay provides a new perspective for understanding tumor immune evasion and offers a foundation for identifying potential therapeutic targets.

## 4 Lactylation and tumor immunity

Lactylation, a novel lysine post-translational modification, tightly links tumor metabolic status with epigenetic regulation and has recently been implicated in shaping the TME and mediating immune evasion (50). Tumor cells generate high levels of lactate through glycolysis, which not only serves as a metabolic byproduct but also influences the function of various immune cells—including T cells, NK cells, TAMs, DCs, and MDSCs-via histone and non-histone lactylation (51). For example, lactate regulates T cell exhaustion phenotypes and immune checkpoint responses through histone lactylation. In NK cells, lactylation induces mitochondrial dysfunction and suppresses cytotoxicity (52). In TAMs, lactate and lactylation promote M2 polarization, enhancing immunosuppression and pro-tumor activity. In DCs and MDSCs, lactylation inhibits antigen presentation and reinforces immunosuppressive signaling, collectively shaping an immunotolerant TME. In summary, lactylation serves as a metabolic–epigenetic bridge in tumor immunity, representing a core mechanism for immune modulation and offering new strategies and potential targets for metabolic-epigenetic-based immunotherapy. As shown in **Table 1**, major lactylation sites in different immune cell populations exhibit distinct regulatory functions within the tumor microenvironment.

**Table 1:** Representative lactylation sites and functional effects in major immune cell types.

Immune cell type	Lactylated site	Mechanistic	Functional
T cells (CD8^+^, CD4^+^, Tregs)	MOESIN K72	Enhances TGF-β–TGFβRI signaling	Promotes Treg activation; contributes to immune evasion and ICI resistance
	Histone H3K18la	Increases transcription of CD39/CD73, POM121	T cell exhaustion; increased PD-L1; reduced CAR-T activity
	RIG-I (site unspecified)	Inhibits NF-κB binding to *Nlrp3*	Suppresses CD8^+^ T cells; enhances Treg activity
NK cells	ROCK1 (site unspecified)	Modulates DRP1 phosphorylation	Mitochondrial fragmentation; reduced cytotoxicity
Tumor-associated macrophages (TAMs)	Histone H3K18la	Activates anti-inflammatory/M2 genes (e.g., *Arg1*)	M2 polarization; immunosuppression; tumor progression
	HMGB1 (multiple sites)	Increases pro-inflammatory and injury signals	Amplifies inflammation or immunosuppression depending on context
	PKM2 (non-histone lactylation)	Activates tetramer formation	Drives M1→M2 transition
	ENSA K63 lactylation	Activates STAT3/CCL2 axis	Recruits TAMs; promotes immunosuppressive TME
Tumor-infiltrating myeloid cells (TIMs)	METTL3 K281/K345	Enhances m6A binding and Jak1 translation	Strengthens STAT3 signaling; ICI resistance
	Histone H3K18la	Upregulates *METTL3* transcription	Promotes immunosuppressive polarization
Dendritic cells (DCs)	Histone lactylation (unspecified sites)	Reduces CD1a, IL-12; increases IL-10	Impaired antigen presentation; reduced T-cell activation
MDSCs	Histone lactylation (unspecified)	Alters metabolic and suppressive gene programs	Enhances immunosuppressive activity
Neutrophils	Histone H3K18la	Upregulates CXCL1/CXCL5, ARG1	Increases recruitment; T-cell suppression; promotes metastasis

### 4.1 T cells

**Figure 1:**
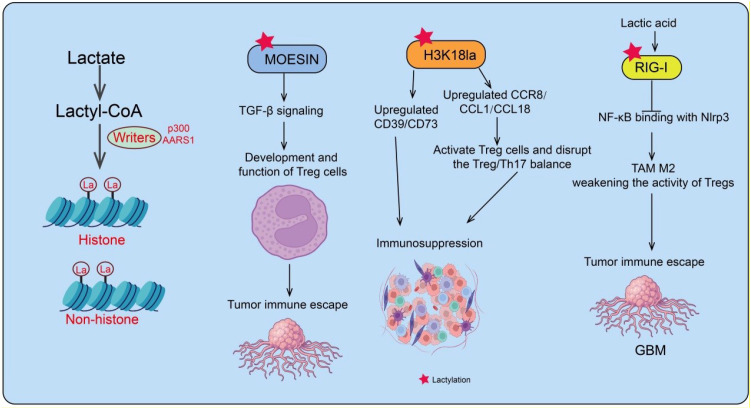
Lactate and histone lactylation-mediated regulation of adaptive immunity and tumor immune escape. Schematic Illustration of Lysine Lactylation on Protein. Histone lactylation and lactate signaling modulate adaptive immune responses. MOESIN promotes TGF-β signaling, supporting Treg cell development and contributing to tumor immune escape. H3K18la upregulates CCR8, CCL1, and CCL18, activating Treg cells and disrupting the Treg/Th17 balance, while also increasing CD39/CD73 expression, collectively enhancing immunosuppression. Lactate produced by E. coli induces RIG-I lactylation, facilitating NF-κB interaction with NLRP3, which polarizes TAMs toward M2 phenotypes and promotes glioblastoma (GBM) immune escape. These mechanisms highlight the role of lactylation in shaping immunosuppressive microenvironments and facilitating tumor progression.

T cells, as central effectors of adaptive immunity, play a pivotal role in anti-tumor responses. Their major subsets include CD8^+^ cytotoxic T lymphocytes (CTLs), CD4^+^ helper T cells (Th), and regulatory T cells (Tregs) (53, 54). Within the TME, CD8^+^ T cells exert direct cytotoxic effects by recognizing tumor antigens, while CD4^+^ T cells contribute to anti-tumor immunity by secreting cytokines and regulating other immune cells (55). Conversely, Tregs maintain immune homeostasis but may suppress anti-tumor immunity, facilitating immune evasion (56). The functions of these T cell subsets are significantly influenced by metabolic dysregulation and lactate accumulation in the TME. High lactate levels not only inhibit T cell proliferation and effector functions but may also induce histone lactylation-mediated transcriptional reprogramming, leading to T cell exhaustion and reduced responsiveness to immune checkpoint inhibitors (ICIs). Mechanistically, Gu et al. reported that lactylation of MOESIN at lysine 72 enhances TGF-β signaling via TGF-β receptor I (but not receptor II) in Tregs, regulating their development and function, thereby promoting tumor immune evasion. Importantly, patients responding to anti-PD-1 therapy exhibit lower MOESIN lactylation than non-responders, suggesting that inhibiting MOESIN lactylation may improve immunotherapy efficacy (57) (**Figure 1**). Additionally, the ectonucleotidases CD39 and CD73 play key roles in establishing an immunosuppressive TME. Sun et al. demonstrated in glioblastoma that lactate promotes H3K18 lactylation (H3K18la) at the promoters of CD39 and CD73, upregulating their expression to enhance immunosuppression (58). Concurrently, lactate increases the expression of CCR8 and its ligands CCL1 and CCL18, activating Tregs and disrupting the Treg/Th17 balance, further strengthening immunosuppression (58) (**Figure 1**). Notably, during chimeric antigen receptor T cell (CAR-T) therapy for glioblastoma, the lactate production inhibitor L-ornithine reduced CD39 and CD73 expression and decreased CCR8 levels in tumor-infiltrating Tregs, thereby promoting CAR-T cell activation. This suggests that targeting lactate production may enhance glioblastoma immunotherapy outcomes. In colorectal cancer liver metastasis (CRLM), Escherichia coli promotes lactate production, inducing RIG-I lactylation (59). This modification inhibits NF-κB binding to the Nlrp3 promoter, downregulating Nlrp3 transcription, which drives macrophage M2 polarization and attenuates Treg immunosuppressive activity and CD8^+^ T cell anti-tumor effects (59). Small-molecule screening identified a compound that inhibits RIG-I lactylation, preventing M2 polarization and enhancing 5-FU sensitivity in CRLM, providing a novel avenue for lactylation-targeted intervention (59) (**Figure 1**). Collectively, lactate and lactylation in the TME exert profound regulatory effects on T cell function, including Treg activation, T cell exhaustion, and responsiveness to immunotherapy. The study found that in non-small cell lung cancer (NSCLC), histone H3K18 lactylation levels are elevated and associated with poor patient prognosis. H3K18 lactylation enhances tumor immune evasion by activating POM121, which promotes MYC nuclear translocation and upregulates PD-L1 expression. Inhibition of glycolysis combined with anti-PD-1 therapy can reverse this process, restore CD8+ T-cell function, and exert significant antitumor effects (60). These findings underscore the close interplay between lactate metabolism and immune regulation and highlight potential targets for optimizing future cancer immunotherapies.

### 4.2 NK cells

**Figure 2:**
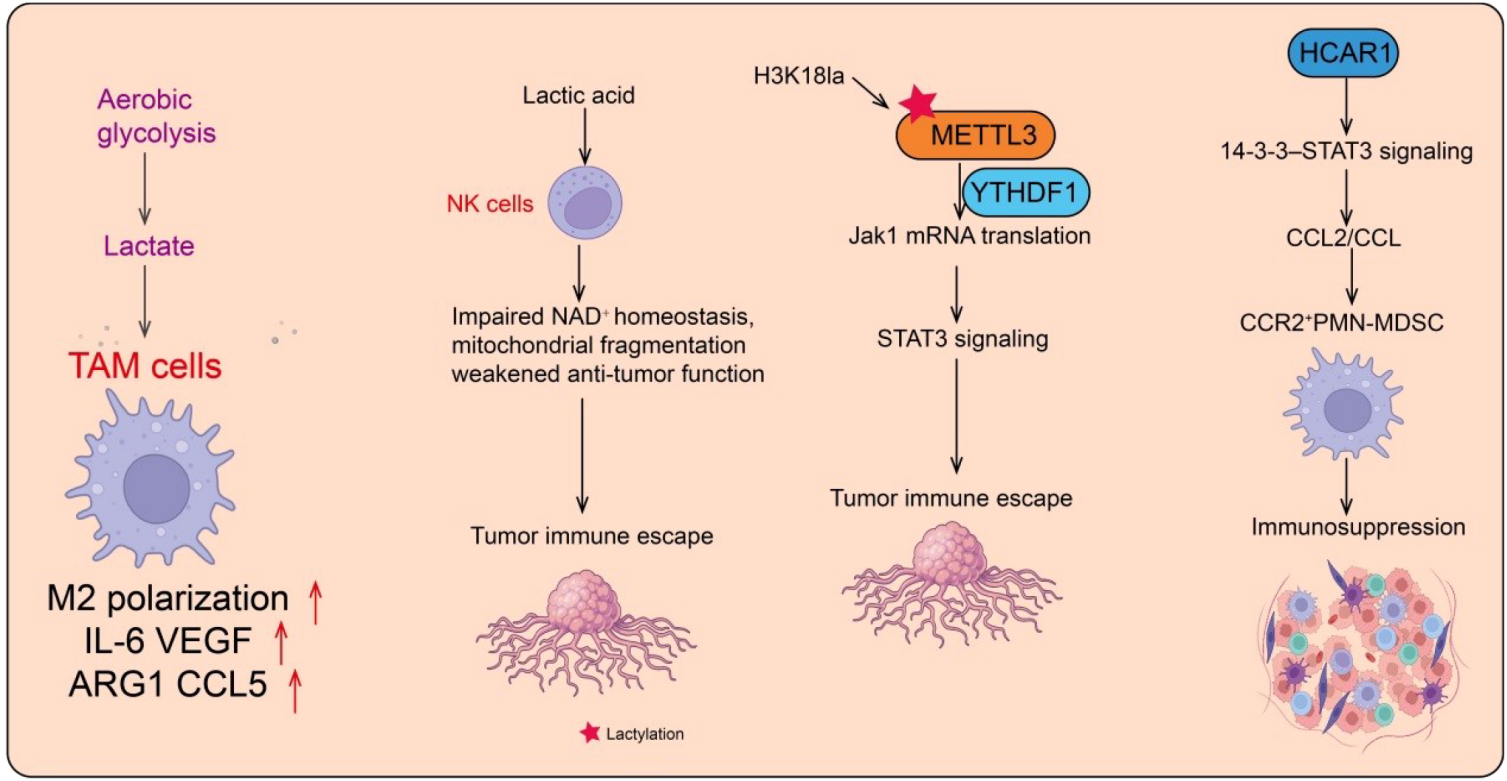
The role of protein lactylation in the regulation of tumor immunity. Aerobic glycolysis in tumor cells produces lactate, which modulates the tumor microenvironment and promotes immune evasion. Lactate induces M2 polarization of tumor-associated macrophages (TAMs), increasing the expression of IL-6, VEGF, ARG1, and CCL5, thereby enhancing tumor immune escape. In NK cells, lactate impairs NAD^+^ homeostasis and mitochondrial integrity, weakening anti-tumor activity. Additionally, histone lactylation (H3K18la) activates METTL3/YTHDF1-mediated STAT3 signaling, facilitating tumor immune escape. Lactate also acts through the HCAR1–14-3-3–STAT3 axis to recruit CCR2^+^ PMN-MDSCs, contributing to immunosuppression within the tumor microenvironment.

NK cells, as critical components of the innate immune system, play a key role in early anti-tumor defense by recognizing and directly killing virus-infected or transformed cells (61, 62). NK cell function depends on the balance between activating and inhibitory receptors and is executed via the release of perforin, granzymes, and interferon-γ (IFN-γ), which not only mediate direct cytotoxicity but also regulate the immune network (62, 63). Within the TME, NK cells contribute to tumor clearance and modulate immune responses through interactions with macrophages, T cells, and dendritic cells. However, metabolites and immunosuppressive signals within the TME frequently impair NK cell function, leading to reduced cytotoxicity and cytokine secretion, thereby facilitating tumor immune evasion (64). Recent studies have revealed that high lactate concentrations in the bone marrow microenvironment induce lysine lactylation in NK cells, with elevated lactylation closely associated with NAD^+^ dysregulation, mitochondrial fragmentation, and attenuated anti-tumor activity (65). Small-molecule intervention strategies demonstrated that combined treatment with nicotinamide riboside (an NAD^+^ precursor) and magnolol (a SIRT3 activator) restores NK cell anti-tumor function by activating the de-lactylase SIRT3 (65). Additionally, this combination regulates lactylation on ROCK1, modulating DRP1 phosphorylation to prevent mitochondrial fragmentation, further enhancing NK cell function. This study first elucidates the mechanism by which TME lactate suppresses NK cell anti-tumor activity through lactylation, offering a novel small-molecule-based intervention strategy (65) (**Figure 2**). Collectively, lactate and lactylation not only inhibit NK cell cytotoxicity but also create new epigenetic targets to enhance anti-tumor immunity.

### 4.3 TAMs

TAMs are among the most abundant immune cell populations within the TME and play dual roles in tumor initiation, progression, and immune evasion (66). TAMs can polarize into M1-like (classically activated, pro-inflammatory, anti-tumor) or M2-like (alternatively activated, immunosuppressive, pro-tumor) phenotypes in response to microenvironmental signals. In most solid tumors, TAMs predominantly adopt an M2-like phenotype, secreting immunosuppressive factors such as IL-10 and TGF-β, promoting angiogenesis and extracellular matrix remodeling, suppressing effector T cell activation, and supporting tumor growth and metastasis (67) (**Figure 2**). Recent studies indicate that lactate and lactylation play critical roles in TAM polarization. High levels of lactate produced by tumor glycolysis promote M2-like polarization via histone lactylation, altering gene expression profiles and establishing an immunosuppressive microenvironment (68, 69). For example, lactate activates mTORC1 and mTORC2-AKT signaling to inhibit the transcription factor TFEB, reduce HIF-2α degradation, and induce macrophage expression of anti-inflammatory and pro-angiogenic genes (70). Lactate also drives M2 polarization via the ERK/STAT3 pathway and, through activation of the GPR81 receptor, downregulates cAMP-PKA signaling to mediate immunosuppression and angiogenesis (71). Tumor-derived exosomes further enhance macrophage glycolysis and lactate production, upregulating PD-L1 expression and reinforcing immune suppression. Conversely, in hepatocellular carcinoma models, D-lactate can repolarize M2 TAMs to an M1 phenotype by inhibiting PI3K-AKT and activating NF-κB, reshaping the immunosuppressive microenvironment (72). The “M1 lactate clock” mechanism facilitates the transition of early M1 macrophages to M2-like characteristics via histone lactylation, a process dependent on p300-mediated p53-associated histone modifications that influence M2 gene promoters such as Arg1 (73). Early M1 macrophages use glycolysis-derived lactate to drive M2 conversion through histone lactylation, while later-stage M2 macrophages undergo metabolic reprogramming via pyruvate entry into the TCA cycle to generate acetyl-CoA, promoting histone acetylation-dependent gene expression, further reinforcing M2 gene programs, immunosuppression, and tumor progression (74). Additionally, lactate-mediated non-histone lactylation activates PKM2 tetramers, suppresses glycolysis, and facilitates M1→M2 transition (75). Exogenous lactate induces HMGB1 lactylation and acetylation, which can exacerbate sepsis, whereas HSPA12A mitigates liver injury by inhibiting HMGB1 lactylation (76). M2 TAMs within the TME simultaneously suppress inflammation and promote tumor proliferation, underscoring the role of histone lactylation in TAM-mediated immune evasion. Specific examples include: in PTEN/p53-deficient prostate cancer, PI3K inhibitors reduce lactate production and H3K18 lactylation in TAMs, enhancing phagocytosis and suppressing tumor growth, whereas Wnt-β-catenin activation restores lactate secretion and H3K18la, leading to ADT/PI3Ki/aPD-1 resistance (77). Tumor lactate upregulates the RARγ promoter via H3K18 lactylation, activating TRAF6-IL-6-STAT3 signaling and promoting tumorigenesis, despite RARγ-TRAF6 interaction normally inhibiting NF-κB and IL-6 (78). NUPR1 upregulation correlates with M2 polarization, increased PD-L1 and SIRPA expression, CD8^+^ T cell suppression, and reduced immunotherapy responsiveness. In MASLD patient liver macrophages, an HK2/glycolysis/H3K18la positive feedback loop exacerbates metabolic imbalance and inflammatory phenotypes; HK2 deletion or HIF-1α inhibition disrupts this cycle (79). In GBM, late-stage monocyte-derived macrophages (MDMs) surpass microglia in suppressing T cell activity, with glycolysis-driven lactate and IL-10 production enhancing immunosuppression via H3K18la; inhibition of glycolysis or lactate production restores T cell activity and delays tumor growth (80). In PDAC, lactate induces ENSA K63 lactylation, activating STAT3/CCL2 signaling, recruiting TAMs, and establishing an immunosuppressive TME (81). In LUAD, NDRG1 stabilizes LDHA, increasing lactate accumulation, promoting M2 polarization, and suppressing CD8^+^ T cell function, further enhanced by H3K18 lactylation (82). In MIBC, PRKN-mediated mitophagy regulates M2 polarization, with H3K18la enhancing PRKN transcription and immunosuppression (83). In PDAC, CTCF induces histone lactylation via the FLG-AS1/HNRNPU/EP300/IGF2BP2 pathway, promoting TAM M2 polarization and reprogramming the TME (84). Recent studies have found that lactate can upregulate TNFR2 expression on Treg cells, thereby enhancing their immunosuppressive function in malignant pleural effusion (MPE). Mechanistically, lactate regulates NF-κB p65 gene transcription through H3K18la, which in turn promotes TNFR2 gene expression and accelerates the progression of MPE. This study elucidates the mechanism by which TNFR2 expression on Treg cells is regulated and its role in the progression of MPE, providing new insights into the epigenetic regulation of tumor development and potential therapeutic strategies targeting lactate metabolism in Treg cells for the treatment of MPE (85). In summary, lactate and lactylation regulate TAM polarization and immunosuppression through multiple signaling and metabolic–epigenetic mechanisms, providing abundant strategies and therapeutic targets for macrophage-targeted tumor immunotherapy.

### 4.4 TIMs

TIMs are critical immune modulators within the TME, with their function significantly influenced by lactylation, particularly in resistance to immune checkpoint inhibitors (ICIs) (86). TIMs utilize histone and protein lactylation to establish immunosuppressive networks, while TME factors reciprocally regulate TIM function. Xiong et al. demonstrated that H3K18la in TIMs upregulates METTL3, promoting m6A modification of Jak1 mRNA. This METTL3-m6A-YTHDF1 axis enhances Jak1 translation on polysomes and STAT3 signaling, reinforcing TME immunosuppression (87) (**Figure 2**). Lactylation of METTL3 at K281 and K345 further augments m6A RNA binding, contributing to immune evasion (87). These findings suggest lactylation regulates TIMs via histone and METTL3 modifications, and METTL3 inhibitors may provide new therapeutic avenues.

### 4.5 Dendritic cells

Dendritic cells (DCs), as central antigen-presenting hubs, play a pivotal role in initiating and regulating antigen-specific anti-tumor immunity (88). DCs capture and process tumor antigens for presentation to T cells while secreting IL-12 and type I interferons (IFN-α/β) to modulate T cell polarization and NK cell activity (89). However, metabolic dysregulation within the TME markedly impairs DC function. Elevated lactate levels and acidic conditions inhibit DC maturation and antigen-presenting capacity, thereby reducing T cell activation efficiency. Lactylation may regulate immune gene expression in DCs through histone or non-histone modifications, directly contributing to immunosuppressive networks (90). For instance, lactate suppresses monocyte differentiation into DCs and diminishes cytokine activity, leading to functional tolerance; it also induces IL-10 production while suppressing TLR-stimulated IL-12 secretion (91). Gottfried et al. demonstrated that *in vitro* DC differentiation in the presence of lactate downregulates CD1a and reduces IL-12 secretion, consistent with the tumor-associated DC (TADC) phenotype observed in melanoma and prostate cancer; inhibition of lactate production restores the DC phenotype (92). Moreover, lactate enhances tryptophan metabolism and kynurenine production in plasmacytoid DCs (pDCs) and FoxP3^+^CD4^+^ Tregs, suppressing IFN-α production and, via GPR81 engagement and intracellular calcium mobilization, blocking IFN-γ output (93). High lactate further inhibits glycolysis, exacerbating pDC dysfunction. In summary, lactate and lactylation suppress DC anti-tumor functions through multiple mechanisms, highlighting the central role of metabolic regulation in tumor immune evasion and providing novel targets for immunotherapeutic intervention (94).

### 4.6 MDSCs

MDSCs are markedly expanded in the TME and serve as key inhibitors of anti-tumor immunity (56, 95). MDSCs promote tumor immune evasion by secreting immunosuppressive cytokines such as IL-10 and TGF-β, depleting arginine and cysteine, and suppressing T cell activation, while also regulating angiogenesis and other immune cell functions to support tumor growth and metastasis (96). Emerging evidence indicates that lactate and C. Elevated lactate levels can alter MDSC metabolic states and, via histone lactylation, regulate gene expression to enhance immunosuppressive activity, highlighting lactylation as a critical metabolic–epigenetic bridge in shaping the immunosuppressive TME. MDSCs comprise pathologically activated neutrophils and monocytes, with polymorphonuclear MDSCs (PMN-MDSCs) being the predominant subset that effectively suppresses T cell proliferation and effector functions (97). In colorectal cancer, high expression of HCAR1 correlates with advanced tumor stage and poorer overall survival. HCAR1 promotes immune suppression by recruiting CCR2^+^ PMN-MDSCs, which inhibit CD8^+^ T cell-mediated anti-tumor immunity. Hcar1 knockout reduces CCR2^+^ PMN-MDSC infiltration, enhances CD8^+^ T cell activation, and decreases tumor burden (98) (**Figure 2**). Mechanistically, HCAR1 activates the 14-3-3–STAT3 pathway, inducing tumor cell expression of CCL2 and CCL7 to recruit CCR2^+^ PMN-MDSCs into the TME, thereby establishing an immunosuppressive network (98). Structural virtual screening identified the FDA-approved drug reserpine as an HCAR1 inhibitor, which reduces CCR2^+^ PMN-MDSC recruitment and enhances CD8^+^ T cell anti-tumor activity. Notably, combining reserpine with PD-1 blockade significantly improved immune responses in a colorectal cancer mouse model, providing a potential strategy for clinical combination therapy (98). In summary, lactate and lactylation regulate MDSC metabolism and immunosuppressive function, playing a central role in tumor immune evasion. Targeting MDSCs and their lactylation mechanisms thus represents a promising direction for future immunotherapeutic interventions.

### 4.7 Neutrophils cells

Neutrophils play critical roles in tumor progression and inflammatory responses through diverse metabolic and epigenetic mechanisms (99). Studies have revealed that GPR37 is highly expressed in CRLM tissues and is associated with poor prognosis (100). GPR37 activates the Hippo pathway to promote LDHA expression and glycolysis, leading to increased H3K18 lactylation, which upregulates CXCL1 and CXCL5 to enhance neutrophil recruitment and tumor metastasis. In addition, hypoxia boosts glucose metabolism and lactate production in neutrophils, inducing histone lactylation that elevates arginase-1 expression and suppresses T-cell function (101). Targeting histone lactylation can block the immunosuppressive activity of CD71^+^ neutrophils, delay tumor progression, and improve the efficacy of immunotherapy. Furthermore, using a zebrafish inflammation model, researchers found that continuous light exposure (LL) significantly increased neutrophil recruitment to injury sites, accompanied by elevated H3K18 lactylation and reactive oxygen species (ROS) levels (101). H3K18 lactylation transcriptionally activates duox, promoting ROS production, while ROS in turn enhance H3K18 lactylation, forming a positive feedback loop that exacerbates inflammatory responses (101). Together, these findings highlight histone lactylation as a central regulator linking neutrophil metabolism, immune suppression, and inflammatory amplification, underscoring the importance of maintaining proper metabolic and environmental rhythms to preserve immune homeostasis.

Protein lactylation plays distinct roles in innate and adaptive immunity, reflecting differences in cellular metabolism and functional outcomes (102). In innate immune cells such as neutrophils and macrophages, lactylation is highly responsive to metabolic cues like glycolysis and hypoxia. For example, H3K18 lactylation in neutrophils upregulates chemokines (CXCL1/5) and arginase-1, promoting immune suppression, excessive recruitment, and modulation of inflammatory responses (103). These modifications act rapidly to link metabolic flux to immediate effector functions. In contrast, in adaptive immune cells such as T cells, lactylation primarily influences gene transcription programs that control differentiation, proliferation, and effector activity. Here, lactylation can indirectly shape immune responses by regulating cytokine production and metabolic checkpoints, thereby modulating long-term immunity. Overall, lactylation serves as a metabolic-epigenetic bridge in both compartments but exerts rapid, effector-oriented effects in innate immunity versus transcriptional, fate-determining roles in adaptive immunity, highlighting its context-dependent functions within the immune system.

### 4.8 Lactylation-modifying enzymes and implications for immunotherapy

Histone and Kla is dynamically regulated by “writers” and “erasers,” linking tumor metabolism to immune regulation. On the writer side, p300 and HBO1 have been identified as lactyltransferases that deposit Kla marks on histones such as H3K18la and H3K9la, directly influencing transcriptional programs associated with PD-L1 expression, M2 macrophage polarization, and Treg stability. Conversely, recent work has uncovered HDAC1–3 and SIRT1–3 as delactylases, capable of removing Kla both *in vitro* and in cells, thereby functioning as negative regulators of lactylation-driven immunosuppression. From a translational perspective, these enzymes represent promising targets to reprogram the TME. In TAMs and MDSCs, inhibiting writers (e.g., p300/HBO1) may suppress lactate-driven immunosuppressive polarization, while activating erasers (e.g., SIRT1/HDAC3) could restore antigen presentation and enhance T-cell priming. In Tregs and exhausted CD8^+^ T cells, manipulating Kla levels may recalibrate transcriptional circuits controlling immune tolerance and effector function. Importantly, combining lactylation modulators with immune checkpoint inhibitors (ICIs, such as anti–PD-1/PD-L1) or cell-based therapies (e.g., CAR-T/NK) offers a rational framework to overcome metabolic resistance mechanisms. For instance, glycolysis blockade or LDH inhibition not only reduces Kla deposition but also synergizes with ICIs to reinvigorate CD8^+^ T-cell cytotoxicity. Overall, targeting the lactylation axis (writers, erasers, and upstream metabolic enzymes) provides a conceptual bridge between tumor metabolism and epigenetic immunoregulation, highlighting novel opportunities for combination immunotherapy.

### 4.9 Lactylation and chemotherapy response

In addition to its profound effects on immunotherapy, emerging evidence indicates that lactylation also plays an important role in shaping tumor responses to chemotherapy (60). Elevated lactate accumulation within the tumor microenvironment can induce histone and non-histone lactylation that reprograms metabolic and inflammatory pathways, thereby influencing chemoresistance. For example, Tumor glycolysis-derived lactate drives lysine lactylation of key DNA repair proteins, contributing to chemotherapy resistance (104). NBS1 K388 lactylation promotes MRN complex formation and homologous recombination, while XLF K288 lactylation enhances Ku80 binding and non-homologous end joining. Inhibition of lactate production or targeted blockade of these lactylation events impairs DNA repair and sensitizes cancer cells to chemotherapeutic agents, highlighting lactylation as a metabolic–epigenetic mechanism underlying chemoresistance (105). Tumor glycolysis-derived lactate promotes XLF K288 lactylation within its Ku-binding motif, enhancing Ku80 binding and recruitment to DNA double-strand breaks, thereby facilitating non-homologous end joining (NHEJ). DNA damage–induced ATM-mediated GCN5 phosphorylation further increases XLF lactylation and NHEJ efficiency. Inhibition of XLF K288 lactylation impairs DNA repair, sensitizes cancer cells to chemotherapy, and synergizes with 5-fluorouracil, highlighting this axis as a potential therapeutic target (106). Thus, lactylation links tumor metabolism to genome stability, representing a key mechanism underlying chemotherapy resistance and a potential therapeutic target. Given that chemotherapeutic agents often depend on intact innate and adaptive immune responses to achieve maximal antitumor effects, lactylation-induced immune suppression may indirectly reduce chemotherapy efficacy. Together, these findings suggest that targeting lactate production or lactylation-related enzymes may not only enhance immunotherapy but also improve chemotherapeutic outcomes, providing a dual pathway for metabolic-epigenetic therapeutic intervention.

## 5 The role of lactylation in tumor immune evasion

Tumor immune evasion is a hallmark of malignant progression, primarily driven by upregulation of immune checkpoint molecules, expansion of immunosuppressive cell populations, and inhibition of effector gene expression. With the recent discovery of lactylation, its critical regulatory role in tumor immune evasion has become increasingly evident. First, lactylation promotes the expression of immune checkpoint molecules through epigenetic mechanisms. For instance, elevated lactate levels enhance histone lactylation in tumor cells, thereby increasing the transcriptional activity of genes such as PD-L1. This process not only suppresses T cell activation and cytotoxicity but may also reduce the efficacy of immune checkpoint inhibitors (ICIs), representing a significant mechanism of immunotherapy resistance (107). Second, lactylation can activate the transcription of multiple immunosuppressive genes. By altering chromatin accessibility, lactylation promotes the expression of genes such as ARG1, VEGF, and IL-10, which contribute to immunosuppression or angiogenesis (108). These gene products remodel the tumor microenvironment to favor the expansion and functional maintenance of immunosuppressive populations, including TAMs, MDSCs, and Tregs, thereby further attenuating anti-tumor immunity (109). Moreover, lactylation often interacts with other metabolic–epigenetic pathways to reinforce immune evasion. It may compete or cooperate with modifications such as acetylation and methylation, collectively modulating the transcriptional state of key immune-related genes. In parallel, lactate metabolism itself contributes to immune suppression by acidifying the microenvironment, inhibiting dendritic cell antigen presentation, and restricting T cell metabolic activity. The combination of these metabolic and epigenetic effects establishes a multilayered immunosuppressive network. In summary, lactylation mediates tumor immune evasion through multiple dimensions: it directly upregulates immune checkpoint molecules to resist immune clearance, and it activates immunosuppressive genes while cooperating with other metabolic modifications to establish a robust immunosuppressive barrier. Elucidating the molecular mechanisms of lactylation in immune escape not only advances our understanding of immunotherapy resistance but also offers new opportunities for developing integrated metabolic–epigenetic therapeutic strategies.

## 6 Significance of targeting lactylation

Lactylation, as a critical interface between metabolism and epigenetic regulation, plays a pivotal role in tumor immune modulation and immune evasion, thereby holding potential therapeutic value. Current strategies targeting lactylation mainly focus on three approaches. First, reducing lactate production to lower lactylation levels. Tumor cells rely on glycolysis for rapid energy supply, and key glycolytic enzymes, such as lactate dehydrogenase A (LDHA) and hexokinase 2 (HK2), have emerged as potential drug targets (110). Inhibiting these enzymes decreases lactate accumulation, thereby reducing lactylation at its source and attenuating tumor-mediated immunosuppression. Second, blocking lactate transmembrane transport can limit its accumulation in the tumor microenvironment. Monocarboxylate transporters (MCTs) are the main carriers mediating lactate transport, and inhibition of MCT1/4 has been shown in multiple tumor models to improve T cell function and enhance the efficacy of ICIs (111). As such, MCTs represent a critical bottleneck linking lactate metabolism and lactylation, offering promising avenues for drug development. Third, targeting the enzymatic machinery underlying lactylation provides opportunities for novel therapeutic interventions. Although the specific “writers” and “erasers” of lactylation remain incompletely defined, current evidence suggests that acetyltransferases such as p300/CBP may mediate lactylation (112). Clarifying the mechanisms of lactylation catalysis and removal could enable direct modulation through small-molecule inhibitors or activators. In addition, lactylation interacts closely with immunotherapy. Studies have demonstrated that lactate accumulation and lactylation upregulate immune checkpoint molecules such as PD-L1, thereby reducing the efficacy of ICIs. Therefore, combining lactylation-targeted strategies with ICIs may overcome certain resistance mechanisms and enhance anti-tumor immunity. Finally, lactylation itself may serve as a novel biomarker for immunotherapy response. Because lactylation levels reflect both tumor metabolic status and the degree of immunosuppression, their detection could provide a valuable tool for predicting and monitoring patient response to immunotherapy. Collectively, lactylation holds dual potential as both a therapeutic target and a biomarker. Although mechanistic studies and clinical translation are still in early stages, lactylation offers a promising foundation for developing integrated metabolic–epigenetic–immune strategies in cancer therapy.

Therapeutic targeting of protein lactylation in cancer is emerging as a promising strategy but faces notable challenges. Approaches include inhibiting lactate production (e.g., blocking glycolysis or LDHA with agents such as oxamate) to reduce the metabolic source of lactylation, interfering with the enzymatic “writers” like p300 to prevent site-specific histone lactylation (113), and enhancing “eraser” activity (HDAC1–3) to remove lactyl marks. In addition, targeting downstream effects—such as blocking lactate-driven histone lactylation with small molecules like isosafrole—has shown potential to reverse immunosuppressive neutrophil activity and sensitize tumors to immunotherapy. However, these strategies face limitations: lactate is a central metabolic intermediate essential for normal tissues, making systemic inhibition prone to toxicity; p300 and HDACs regulate multiple epigenetic marks beyond lactylation, raising concerns about off-target effects (114); and the non-enzymatic D-lactylation pathway is more difficult to control due to its spontaneous nature. Therefore, future therapies may require precision delivery, context-specific inhibitors, or combination approaches to selectively modulate lactylation in the tumor microenvironment while preserving physiological metabolic functions.

Clarifying the therapeutic pathways for targeting protein lactylation provides new opportunities to overcome tumor immune resistance, including cases where PD-1 inhibitors are ineffective (115). Upstream inhibition aims to cut off the lactate source by using LDHA inhibitors (e.g., FX11) or monocarboxylate transporter (MCT) blockers to reduce lactate production and secretion. Process interference focuses on directly modulating lactylation levels through p300 inhibitors to prevent lactyl group transfer or by activating delactylating enzymes such as HDACs or SIRT3 to accelerate removal of lactyl marks (116). Microenvironment neutralization employs strategies such as bicarbonate-based nanoparticles to buffer the acidic tumor milieu, thereby disrupting lactate-driven signaling. Finally, combination therapy integrates these approaches with immune checkpoint blockade to synergistically reverse immunosuppression, transform “cold” tumors into “hot” ones, and ultimately enhance patient responses to immunotherapy.

## 7 Conclusion and perspectives

Lactylation, a recently discovered class of post-translational modification, reveals a novel role for lactate in tumor immunity beyond being a mere metabolic byproduct. By linking metabolic homeostasis with epigenetic regulation, lactylation exerts critical functions in the tumor microenvironment, influencing not only tumor cell gene expression and adaptive evolution but also shaping immune cell behavior, thereby playing a key role in tumor immune evasion and immunotherapy responses. Current studies indicate that lactylation is particularly important in TAMs, driving their polarization toward an immunosuppressive phenotype. However, the specific roles of lactylation in T cells, dendritic cells, myeloid-derived suppressor cells, and other immune populations remain underexplored, limiting a comprehensive understanding of its function within the tumor immune network. Several scientific questions remain unresolved. First, the enzymatic mechanisms of lactylation are not fully elucidated; while p300/CBP acetyltransferases may be involved, the existence and function of potential “delactylases” are still unclear. Second, the reversibility and dynamic regulation of lactylation across different cell types require further investigation. Additionally, lactylation does not act in isolation but interacts with other epigenetic modifications, such as m6A RNA methylation, protein glycosylation, and histone acetylation or methylation, either competitively or cooperatively (117), to regulate key gene transcription and cellular functions. This multilayered crosstalk adds complexity to tumor immune regulation and suggests that therapeutic interventions targeting lactylation must consider potential off-target effects on other pathways. Third, selective targeting of lactylation without perturbing normal metabolism or other epigenetic modifications remains a major challenge for clinical translation. Nonetheless, the discovery of lactylation has opened new avenues in tumor immunology. With advances in detection technologies and mechanistic research, lactylation has the potential to serve as a biomarker for immunotherapy response and provide a foundation for integrated metabolic–epigenetic–immune therapeutic strategies. Future studies integrating multi-omics analyses, animal models, and clinical sample validation are expected to fully elucidate the complex network of lactylation and its interplay with other epigenetic modifications in tumor immunity, paving the way from basic research to clinical application.
